# Protective role of *Scoparia dulcis *plant extract on brain antioxidant status and lipidperoxidation in STZ diabetic male Wistar rats

**DOI:** 10.1186/1472-6882-4-16

**Published:** 2004-11-02

**Authors:** Leelavinothan Pari, Muniappan Latha

**Affiliations:** 1 Department of Biochemistry, Faculty of Science, Annamalai University, Annamalai Nagar, Tamil Nadu-608 002, India

## Abstract

**Background:**

The aim of the study was to investigate the effect of aqueous extract of *Scoparia dulcis *on the occurrence of oxidative stress in the brain of rats during diabetes by measuring the extent of oxidative damage as well as the status of the antioxidant defense system.

**Methods:**

Aqueous extract of *Scoparia dulcis *plant was administered orally (200 mg/kg body weight) and the effect of extract on blood glucose, plasma insulin and the levels of thiobarbituric acid reactive substances (TBARS), hydroperoxides, superoxide dismutase (SOD), catalase (CAT), glutathione peroxidase (GPx), glutathione-S-transferase (GST) and reduced glutathione (GSH) were estimated in streptozotocin (STZ) induced diabetic rats. Glibenclamide was used as standard reference drug.

**Results:**

A significant increase in the activities of plasma insulin, superoxide dismutase, catalase, glutathione peroxidase, glutathione-S-transferase and reduced glutathione was observed in brain on treatment with 200 mg/kg body weight of *Scoparia dulcis *plant extract (SPEt) and glibenclamide for 6 weeks. Both the treated groups showed significant decrease in TBARS and hydroperoxides formation in brain, suggesting its role in protection against lipidperoxidation induced membrane damage.

**Conclusions:**

Since the study of induction of the antioxidant enzymes is considered to be a reliable marker for evaluating the antiperoxidative efficacy of the medicinal plant, these findings suggest a possible antiperoxidative role for *Scoparia dulcis *plant extract. Hence, in addition to antidiabetic effect, *Scoparia dulcis *possess antioxidant potential that may be used for therapeutic purposes.

## Background

The neurological consequences of diabetes mellitus in the Central Nervous System (CNS) are now receiving greater attention. Cognitive deficits, along with morphological and neurochemical alterations illustrate that the neurological complications of diabetes are not limited to peripheral neuropathies [1]. The central complications of hyperglycemia also include the potentiation of neuronal damage observed following hypoxic/ischemic events, as well as stroke [2]. Glucose utilization is decreased in the brain during diabetes [2], providing a potential mechanism for increased vulnerability to acute pathological events.

Oxidative stress, leading to an increased production of reactive oxygen species (ROS), as well as lipidperoxidation, is increased in diabetes [3] and also by stress in euglycemic animals [4]. Similarly, oxidative damage in rat brain is increased by experimentally induced hyperglycemia [5]. Under experimental conditions, hyperglycemia dramatically increases neuronal alterations and glial cell damage caused by temporary ischaemia [6]. Several lines of evidence indicate that the modified oxidative state induced by chronic hyperglycemia [7] may contribute to nervous tissue damage: free radical species impair the central nervous system, attacking neurons and schwann cells [8] and the peripheral nerves [9]. Due to their high polyunsaturated lipid content, schwann cells and axons are particularly sensitive to oxygen free radical damage: lipidperoxidation may increase cell membrane rigidity and impair cell function.

Increases in superoxide production are observed in the serum of Type 1 diabetic patients and was reduced with improved glycemic control [10]. Lipidperoxidation products are also increased in the brains of Type 1 diabetic rats [11] and Type 2 diabetic mice [8]. Diabetes and stress mediated increases in oxidative stress, as well as decreases in antioxidant activity, may make the brain more vulnerable to subsequent pathological events.

Nowadays, the use of complementary/alternative medicine and especially the consumption of botanicals have been increasing rapidly worldwide, mostly because of the supposedly less frequent side effects when compared to modern western medicine [12]. *Scoparia dulcis *L (Scrophulariacae), a folk-medicinal plant known as sweet broomweed, has been used as a remedy for diabetes mellitus in India [13] and for hypertension in Taiwan [14,15]. A number of active principles from *Scoparia dulcis *include scoparic acid A, scoparic acid B and scoparic acid D [16], scopadulcic acid A and B, scopadulciol [17] and Scopadulin [18] that have been identified as contributor to the observed medicinal effect of the plant. Among them, scopadulcic acid B (SDB) and scopadulciol (SDC) were found to be unique biomolecules with inhibitory effects on replication of herpes simplex virus type 1 (HSV-1) [16], gastric proton pump and bone resorption stimulated by parathyroid hormone (PTH) [18]. In addition, SDB showed antitumour promoting activities [17]. Because of their unique carbon skeleton and many sided biological activities, they were paid much attention as chemical synthetic targets by organic synthetic chemists. In a previous study, Nath (1943) studied the antidiabetic effect of *Scoparia dulcis *and obtained a glycoside, amellin from fresh plant and reported that it brought relief in other complications accompanied with diabetes (ie., pyorrhoea, retinopathy, joint pain, susceptibility to cold etc.) within a very short period [19].

Administration of *Scoparia dulcis *to STZ diabetic rats led to reduction in blood glucose [20]. In Recent studies on this plant, we have demonstrated a defective metabolism of lipid peroxides in tissues (liver, kidney and brain) of STZ diabetic rats [21] for 3 weeks treatment. Since increases in oxidative stress are associated with both long standing diabetes and stress, the present investigation was to assess the antioxidant efficacy of *Scoparia dulcis *in STZ diabetic rats after 6 weeks treatment and the effect produced by *Scoparia dulcis *was compared with Glibenclamide.

## Methods

### Animals

Adult male albino Wistar rats (8 weeks), weighing 180–200 g bred in the Central Animal House, Rajah Muthiah Medical College, Annamalai University, were used. All animal experiments were approved by the ethical committee (Vide. No: 73, 2002), Annamalai University and were in accordance with the guidelines of the National Institute of Nutrition, Indian Council of Medical Research, Hyderabad, India. The animals were fed *ad libitum *with normal laboratory pellet diet (Hindustan Lever Ltd., India) and water. Animals were maintained under a constant 12 h light and dark cycle and at an environmental temperature of 21–23°C.

### Drugs and chemicals

All the drugs and biochemicals used in this experiment were purchased from Sigma Chemical Company Inc., St Louis, Mo, USA. The chemicals were of analytical grade.

### Plant material

Whole plants of *Scoparia dulcis *L. (40 – 60 cm in height) were collected from Neyveli, Cuddalore District, Tamil Nadu, India in September 2001. The plant was identified and authenticated at the Herbarium of Botany Directorate in Annamalai University. A voucher specimen (No.3412) was deposited in the Botany Department of Annamalai University.

### Preparation of *Scoparia dulcis *plant extract (SPEt)

Five-hundred grams of fresh whole *Scoparia dulcis *plants were extracted with 1.5 l of water by the method of continuous hot extraction at 60°C for 6 h according to Jain (1968) and the filtrate was concentrated at 40°C to constant weight in a rotavapor apparatus (Buchi Labortechnik AG, Switzerland). The residue collected (yield 31 g) were thick paste, green in color and gumaceaous in nature and stored at -20°C, when needed the extract was dissolved in sterile water and used in the investigation [22].

### Induction of experimental diabetes

STZ, freshly prepared in 10 mmol/l citrate buffer, pH 4.5, was injected to experimental animals (30 rats) intraperitoneally at a dose of 45 mg/kg body weight [23]. 48 h after STZ administration, rats with moderate diabetes having glycosuria and hyperglycemia (i.e with blood glucose of 200 – 300 mg/dl) were taken for the experiment.

### Experimental design

In the experiment, a total of 30 rats (18 diabetic surviving rats, 12 normal rats) were used. The rats were divided into 5 groups of 6 rats each. Group 1: Normal rats. Group 2: Normal rats given *Scoparia dulcis *plant extract (SPEt) (200 mg/kg body weight) in aqueous solution daily using an intragastric tube for 6 weeks. Group 3: Diabetic control rats. Group 4: Diabetic rats given SPEt (200 mg/kg body weight) [21] in aqueous solution daily using an intragastric tube for 6 weeks. Group 5: Diabetic rats given Glibenclamide (600 μg/kg body weight) in aqueous solution daily using an intragastric tube for 6 weeks [24].

All doses were started after 48 h STZ injection. No detectable irritation or restlessness was observed after each drug or vehicle administration. No noticeable adverse effect (i.e., respiratory distress, abnormal locomotion and catalepsy) was observed in any animals after the drug administration. Blood samples were drawn at weekly intervals till the end of study (ie. 6 weeks). At the end of 6^th ^week, all the rats were killed by decapitation (Pentobarbitone sodium) anaesthesia (60 mg/kg). Blood was collected in two different tubes (i.e.,) one with anticoagulant – potassium oxalate and sodium fluoride for plasma and another without anticoagulant for serum separation. Plasma and serum were separated by centrifugation. Whole Brain was immediately dissected out, washed in ice cold saline to remove the blood. The brains were weighed and 10% tissue homogenate was prepared with 0.025 M Tris – HCl buffer, pH 7.5. After centrifugation at 200 rpm for 10 min, the clear supernatant was used to measure thiobarbituric acid reactive substances (TBARS), hydroperoxides and GPx activity. For the assay of SOD, CAT, GST and GSH, the brains were weighed and 10% homogenate was prepared with 0.2 M, phosphate buffer pH 8.0. After centrifugation, the clear supernatant was used for the assay of enzyme activities.

### Biochemical analysis

#### Estimation of blood glucose and plasma insulin

Blood glucose was determined by the O-toluidine method [25]. Plasma insulin was assayed by ELISA, using Boeheringer-Mannheim Kit with a Boeheringer analyser ES300.

#### Estimation of lipid peroxidation

Lipid peroxidation in brain was estimated colorimetrically by thiobarbituric acid reactive substances TBARS and hydroperoxides by the method of Niehius and Samuelsson [26] and Jiang et al. [27], respectively. In brief, 0.1 ml of tissue homogenate (Tris-Hcl buffer, pH 7.5) was treated with 2 ml of (1:1:1 ratio) TBA-TCA-HCl reagent (thiobarbituric acid 0.37%, 0.25 N HCl and 15% TCA) and placed in water bath for 15 min, cooled. The absorbance of clear supernatant was measured against reference blank at 535 nm.

0.1 ml of tissue homogenate was treated with 0.9 ml of Fox reagent (88 mg butylated hydroxytoluene (BHT), 7.6 mg xylenol orange and 9.8 mg ammonium ion sulphate were added to 90 ml of methanol and 10 ml 250 mM sulphuric acid) and incubated at 37°C for 30 min. The color developed was read at 560 nm colorimetrically. Hydroperoxides was expressed as mM/100g tissue.

#### Assay of catalase (CAT) and superoxide dismutase (SOD)

CAT was assayed colorimetrically at 620 nm and expressed as μmoles of H_2_O_2 _consumed/min/mg protein as described by Sinha [28]. The reaction mixture (1.5 ml, vol) contained 1.0 ml of 0.01 M pH 7.0 phosphate buffer, 0.1 ml of tissue homogenate (supernatant) and 0.4 ml of 2 M H_2_O_2_. The reaction was stopped by the addition of 2.0 ml of dichromate-acetic acid reagent (5% potassium dichromate and glacial acetic acid were mixed in 1:3 ratio).

SOD was assayed utilizing the technique of Kakkar et al. [29] based on inhibition of the formation of nicotinamide adenine dinucleotide, phenazine methosulfate and amino blue tetrazolium formazan. A single unit of enzyme was expressed as 50% inhibition of NBT (Nitroblue tetrazolium) reduction/min/mg protein.

#### Determination of glutathione peroxidase (GPx) and reduced glutathione (GSH)

GPx activity was measured by the method described by Rotruck et al. [30]. Briefly, reaction mixture contained 0.2 ml of 0.4 M Tris-HCl buffer pH 7.0, 0.1 ml of 10 mM sodium azide, 0.2 ml of tissue homogenate (homogenised in 0.4 M, Tris-HCl buffer, pH 7.0), 0.2 ml glutathione, 0.1 ml of 0.2 mM hydrogen peroxide. The contents were incubated at 37°C for 10 min. The reaction was arrested by 0.4 ml of 10% TCA, and centrifuged. Supernatant was assayed for glutathione content by using Ellmans reagent (19.8 mg of 5,5'-dithiobisnitro benzoic acid (DTNB) in 100 ml of 0.1% sodium nitrate).

GSH was determined by the method of Ellman [31]. 1.0 ml of supernatant was treated with 0.5 ml of Ellmans reagent and 3.0 ml of phosphate buffer (0.2 M, pH 8.0). The absorbance was read at 412 nm. Glutathione peroxidase activity was expressed as μg of GSH consumed/min/mg protein and reduced glutathione as mg/100g of tissue.

#### Determination of glutathione-S-transferase (GST)

The GST activity was determined spectrophotometrically by the method of Habig et al. [32]. The reaction mixture (3 ml) contained 1.0 ml of 0.3 mM phosphate buffer (pH 6.5), 0.1 ml of 30 mM 1-chloro-2, 4-dinitrobenzene (CDNB) and 1.7 ml of double distilled water. After pre-incubating the reaction mixture at 37°C for 5 min, the reaction was started by the addition of 0.1 ml of tissue homogenate and 0.1 ml of glutathione as substrate. The absorbance was followed for 5 min at 340 nm. Reaction mixture without the enzyme was used as blank. The activity of GST is expressed as μmoles of GSH-CDNB conjugate formed/min/mg protein using an extinction coefficient of 9.6 mM^-1 ^cm^-1^.

#### Estimation of protein

Protein was determined by the method of Lowry et al. [33] using Bovine Serum Albumin (BSA) as standard, at 660 nm.

### Statistical analysis

All data were expressed as mean ± SD of number of experiments (n = 6). The statistical significance was evaluated by one-way analysis of variance (ANOVA) using SPSS version 7.5 (SPSS, Cary, NC, USA) and the individual comparison were obtained by Duncan's Multiple Range Test (DMRT). A value of p < 0.05 was considered to indicate a significant difference between groups [34].

## Results

Table 1 shows the level of blood glucose and plasma insulin in normal and experimental groups. The level of blood glucose was significantly increased whereas the level of plasma insulin was significantly decreased in diabetic control rats. Oral administration of SPEt and glibenclamide to diabetic rats significantly reversed all these changes to near normal levels.

**Table 1:** Effect of *Scoparia dulcis *on blood glucose and plasma insulin in normal and experimental rats

**Groups**	**Fasting blood glucose (mg/dl)**	**Plasma insulin (μu/ml)**
Normal	84 ± 4^a^	12 ± 1^a^
Normal + SPEt (200 mg/kg)	77 ± 3^a^	15 ± 1^b^
Diabetic control	270 ± 15^b^	4 ± 0.3^c^
Diabetic + SPEt (200 mg/kg)	98 ± 3^c^	11 ± 4^d^
Diabetic + Glibenclamide (600 μg/kg)	114 ± 9^d^	9 ± 0.5^e^

Values are given as mean ± SD from 6 rats in each group.

Values not sharing a common superscript letter differ significantly at p < 0.05 (DMRT); Duncan Procedure; Ranges for the level: 2.95, 3.09, 3.20, 3.22.

Table 2 illustrates markers of lipidperoxidatioon namely, TBARS and hydroperoxides from brain of normal and experimental rats. The levels of TBARS and hydroperoxides were significantly increased in diabetic control rats. Administration of SPEt to diabetic rats significantly decreased the levels of lipidperoxidative markers. Treatment of normal rats with SPEt did not show significant changes in lipidperoxidation. The effect produced by SPEt was significant than glibenclamide.

**Table 2:** Change in the levels of brain TBARS and hydroperoxides in normal and experimental rats

**Groups**	**TBARS (mM/100g tissue)**	**Hydroperoxides (mM/100g tissue)**
Normal	1.10 ± 0.08^a^	113.20 ± 4.10^a^
Normal + SPEt (200 mg/kg)	0.90 ± 0.05^a^	108.70 ± 2.20^b^
Diabetic control	1.85 ± 0.07^b^	130.90 ± 1.50^c^
Diabetic + SPEt (200 mg/kg)	1.18 ± 0.06^c^	117.22 ± 3.26^d^
Diabetic + Glibenclamide (600 μg/kg)	1.32 ± 0.06^d^	120.40 ± 4.05^e^

Values are given as mean ± SD for 6 rats in each group.

Values not sharing a common superscript letter differ significantly at p < 0.05 (DMRT).

Duncan procedure, Range for the level 2.95, 3.09, 3.20, 3.22.

For studying the effect of SPEt on antioxidant status, the activities of enzymic antioxidants SOD, CAT, GPx, GST and non-enzymic antioxidant GSH were measured (Table 3 and 4). The activities of enzymic and the levels of non-enzymic antioxidant were significantly decreased in diabetic control rats. They presented significant increases in diabetic rats treated SPEt. Administration of SPEt to normal rats increased the antioxidants levels with no significant differences. The effect produced by SPEt was comparable with that of glibenclamide.

**Table 3:** Changes in activities of catalase and superoxide dismutase in brain of normal and experimental rats

**Groups**	**Catalase (Units^A^/mg protein)**	**Superoxide dismutase (Units^B^/mg protein)**
Normal	3.12 ± 0.29^a^	7.75 ± 0.38^a^
Normal + SPEt (200 mg/kg)	4.00 ± 0.20^b^	7.05 ± 0.28^a^
Diabetic control	0.86 ± 0.05^c^	5.17 ± 0.30^b^
Diabetic + SPEt (200 mg/kg)	2.75 ± 0.20^d^	7.32 ± 0.46^c^
Diabetic + Glibenclamide (600 μg/kg)	1.98 ± 0.11^e^	6.32 ± 0.30^d^

Values are given as mean ± SD for 6 rats in each group.

Values not sharing a common superscript letter differ significantly at p < 0.05 (DMRT).

Duncan procedure, Range for the level 2.95, 3.09, 3.20, 3.22.

A – μmole of H_2_O_2 _consumed/minute.

B – One unit of activity was taken as the enzyme reaction, which gave 50% inhibition of NBT reduction in one minute.

**Table 4:** Changes in activities of glutathione peroxidase, glutathione-S-transferase and the levels of reduced glutathione in brain of normal and experimental rats

**Groups**	**Glutathione peroxidase (Units^A^/mg protein)**	**Glutathione-S-transferase (Units^B^/mg protein)**	**Reduced glutathione (mg/100g tissue)**
Normal	3.4 1± 0.20^a^	5.62 ± 0.28^a^	35.19 ± 2.21^a^
Normal + SPEt (200 mg/kg)	3.80 ± 0.18^a^	5.90 ± 0.28^a^	37.12 ± 2.14^a^
Diabetic control	1.01 ± 0.05^b^	0.81 ± 0.02^b^	15.20 ± 1.44^b^
Diabetic + SPEt (200 mg/kg)	2.62 ± 0.15^c^	2.10 ± 0.13^c^	26.02 ± 2.01^c^
Diabetic + Glibenclamide (600 μg/kg)	1.97 ± 0.12^d^	2.04 ± 0.13^c^	25.50 ± 2.10^c^

Values are given as mean ± SD for 6 rats in each group.

Values not sharing a common superscript letter differ significantly at p < 0.05 (DMRT).

Duncan procedure, Range for the level 2.95, 3.09, 3.20, 3.22.

A – μg of GSH consumed/min.

B – μmoles of CDNB – GSH conjugate formed/min.

## Discussion

This work is one of a series of studies showing that chronic hyperglycemia causes an imbalance in the oxidative status of the nervous tissue and that the resulting free radicals damage the brain through a peroxidative mechanism. The STZ diabetic rat serves as an excellent model to study the molecular, cellular and morphological changes in brain induced by stress during diabetes [7]. Under normal conditions, the generation of free radicals or of active species in the brain, as in other tissues, is maintained at extremely low levels [4]. Diabetes also contributes to cerebrovascular complications, reductions in cerebral blood flow, disruption of the blood brain barrier and cerebral edema [5]. All of these neurochemical and neurophysiological changes ultimately contribute to the long-term complications associated with diabetes, including morphological abnormalities, cognitive impairments and increased vulnerability to pathophysiological event [6].

In the present study, treatment with aqueous extract of *Scoparia dulcis *showed significant antihyperglycemic activity. The antihyperglycemic activity of this plant may be, at least in part, through release of insulin from the pancreas in view of the measured increase in the plasma insulin concentrations.

Earlier studies in this lab have demonstrated a defective metabolism of lipid peroxides in other tissues of diabetic animal [35,36]. TBARS and hydroperoxides (lipid peroxidative markers) showed high lipidperoxidation. This may be because; the brain contains relatively high concentration of easily peroxidizable fatty acids [37]. In addition, it is known that certain regions of the brain are highly enriched in iron, a metal that, in its free form, is catalytically involved in production of damaging oxygen free radical species [38]. It has been suggested that free radical species responsible for STZ toxicity is the hydroxyl radical, formed via the metal catalyzed Haber-weiss reaction or Fenton reaction. In this process, the ferric iron is reduced by superoxide, with subsequent oxidation of ferrous iron by H_2_O_2 _forming hydroxyl radical:

Fe^3+ ^+ O_2_^•- ^→ Fe^2+ ^+ O_2_

Fe^2+ ^+ H_2_O_2 _→ Fe^3+ ^+ OH* + OH^-^

The destruction of superoxide radical or H_2_O_2 _by SOD or CAT would ameliorate STZ toxicity, as would substances able to scavenge the hydroxyl radical [39,40]. Vulnerability of brain to oxidative stress induced by oxygen free radicals seems to be due to the fact that, on one hand, the brain utilizes about one fifth of the total oxygen demand of the body and on the other, that it is not particularly enriched, when compared with other organs, in any of the antioxidant enzymes. Relatively low levels of these enzymes may be responsible in part for the vulnerability of this tissue [41].

The altered balance of the antioxidant enzymes caused by decrease in CAT, SOD, GPx, GST and GSH activities may be responsible for the inadequacy of the antioxidant defenses in combating ROS mediated damage. The decreased activities of CAT and SOD may be a response to increased production of H_2_O_2 _and O_2 _by the autoxidation of glucose and non-enzymatic glycation [5]. These enzymes have been suggested as playing an important role in maintaining physiological levels of oxygen and hydrogen peroxide by hastening the dismutation of oxygen radicals and eliminating organic peroxides and hydroperoxides generated from inadvertent exposure to STZ [42]. Treatment with SPEt increased the activity of enzymes and may help to control free radicals, as *Scoparia dulcis *has been reported to be rich in alkaloids and terpenoids [16-18,43,44], well-known antioxidants, which scavenge the free radicals generated during diabetes. The increase in SOD activity may protect CAT and GPx against inactivation by O_2_^•- ^anions as these anions have been shown to inactivate CAT and GPx [45].

Under in vivo conditions, GSH acts as an antioxidant and its decrease was reported in diabetes mellitus [46]. We have observed significant decrease in GSH levels in brain during diabetes. The decrease in GSH levels represents increased utilization due to oxidative stress [47]. The depletion of GSH content may also lower the GST activity [48]. Depression in GPx activity was also observed brain of diabetic rats. GPx has been shown to be an important adaptive response to condition of increased peroxidative stress [46]. The increased GSH content in the brain of the rats treated with SPEt and glibenclamide may be a factor responsible for inhibition of lipidperoxidation. The elevated level of GSH protects cellular proteins against oxidation through glutathione redox cycle and also directly detoxifies reactive oxygen species generated from exposure to STZ [48]. The significant increase in GSH content and GSH dependent enzymes GPx and GST in diabetic rats treated with SPEt indicates an adaptive mechanism in response to oxidative stress.

Significantly lower levels of lipid peroxides in brain of SPEt treated diabetic rats and increased activities of enzymic and non-enzymic antioxidants in brain suggest that the extract reduce oxidative stress by quenching free radicals. Terpenoids and alkaloids were reported to have free radical scavenging activity and antioxidant capacity in diabetes [49,50]. SPEt was reported to be rich in an alkaloid-6-methoxybenzoxazolinone [51] and terpenoids such as scoparic acids A, B, C and scopadulcic acids A and B [16-18], which may be responsible for scavenging free radicals liberated by STZ and thus enhance both enzymic and non-enzymic antioxidants in diabetic rats treated with SPEt. Any compound, natural or synthetic with antioxidant properties that might contribute towards the partial or total alleviation of this damage may have a significant role in the treatment of diabetes mellitus. The antioxidant responsiveness mediated by *Scoparia dulcis *may be anticipated to have biological significance in eliminating reactive free radicals that may otherwise affect the normal cell functioning. The disfunctioning of these antioxidant enzymes has been implicated in several disorders including rheumatoid arthritis, reperfusion injury, cardiovascular diseases, immune injury as well as diabetes mellitus [52].

It may be concluded that in diabetes, brain tissue was more vulnerable to oxidative stress and showed increased lipidperoxidation. The above observation shows that the aqueous extract of *Scoparia dulcis *plant possesses antioxidant activity, which could exert a beneficial action against pathological alterations caused by the presence of free radicals in STZ diabetes.

## Conclusions

The brain exhibits numerous morphological and functional alterations during diabetes. Oxidative stress, a factor implicated in the pathogenesis of diabetic complications may contribute towards some of these alterations. Treatment of diabetic rats with *Scoparia dulcis *plant extract significantly decreased the lipidperoxidation and significantly increased the antioxidant status. Since the study of induction of the antioxidant enzymes is considered to be a reliable marker for evaluating the antioxidant efficacy of the medicinal plant, these findings are suggestions of possible antioxidant role played by *Scoparia dulcis *plant extract in addition to its antidiabetic effect.

## Competing interests

The author(s) declare that they have no competing interests

## Authors' contributions

LP – supervised the design and co-ordination of the study

ML – Practically conducted the design of the study and drafted the manuscript.

## Pre-publication history

The pre-publication history for this paper can be accessed here:


